# Prospects for a Statistical Theory of LC/TOFMS Data

**DOI:** 10.1007/s13361-012-0340-z

**Published:** 2012-02-29

**Authors:** Andreas Ipsen, Timothy M. D. Ebbels

**Affiliations:** Biomolecular Medicine, Department of Surgery and Cancer, Faculty of Medicine, Sir Alexander Fleming Building, Imperial College London, London, SW7 2AZ UK

**Keywords:** Liquid chromatography, Mass Spectrometry, Time-of-Flight, Statistics, Statistical inference, Poisson, Time-to-digital converter, Maximum likelihood estimation, Test of hypothesis, Likelihood ratio test

## Abstract

The critical importance of employing sound statistical arguments when seeking to draw inferences from inexact measurements is well-established throughout the sciences. Yet fundamental statistical methods such as hypothesis testing can currently be applied to only a small subset of the data analytical problems encountered in LC/MS experiments. The means of inference that are more generally employed are based on a variety of heuristic techniques and a largely qualitative understanding of their behavior. In this article, we attempt to move towards a more formalized approach to the analysis of LC/TOFMS data by establishing some of the core concepts required for a detailed mathematical description of the data. Using arguments that are based on the fundamental workings of the instrument, we derive and validate a probability distribution that approximates that of the empirically obtained data and on the basis of which formal statistical tests can be constructed. Unlike many existing statistical models for MS data, the one presented here aims for rigor rather than generality. Consequently, the model is closely tailored to a particular type of TOF mass spectrometer although the general approach carries over to other instrument designs. Looking ahead, we argue that further improvements in our ability to characterize the data mathematically could enable us to address a wide range of data analytical problems in a statistically rigorous manner.

## Introduction

The use of liquid chromatography time-of-flight mass spectrometry (LC/TOFMS) for biological research has undergone considerable growth over recent years, which has prompted the development of a large number of bioinformatics techniques to facilitate the analysis of the resulting data. Several comprehensive software packages [1–5] are now available, which provide extensive tools for the pre-processing and analysis of LC/TOFMS data, and LC/MS data in general. Yet despite these efforts, the task of extracting useful information from the large data-sets produced through LC/TOFMS assays of complex biological mixtures such as blood or urine remains a central bottleneck to much of the work being carried out in proteomics and metabolomics. Moreover, nothing in the way of a consensus has been established as to how to best approach the development of the required bioinformatics techniques, as the theoretical basis on which they are built is currently rather thin.

There are a large number of pre-processing techniques that are routinely applied to LC/TOFMS data as part of their analysis, and these come in roughly two classes: those applied by the manufacturer’s software prior to the data being output to file, and those applied subsequently by the analyst, often through software packages such as those cited above. The former class includes fundamental methods of data compression, as well as algorithms that compensate for detector saturation, and which may effectively be part of the physical measurement process [6]. The latter include baseline subtraction, smoothing, and feature extraction, as well as methods for standardizing data from different experiments, such as normalization and chromatographic retention time alignment. The pre-processing is followed by the inferential stage of the analysis, which often involves the identification of unknown compounds and, if multiple biological samples are involved, finding differences between groups of samples or examining the relationship between the mass spectra and a continuous parameter of interest, such as age.

It is clear that the pre-processing will have substantial effects on the data and, consequently, on the inferences drawn in downstream analyses [7–9]. Therefore, the choice of pre-processing techniques and the manner in which they are applied is extremely important. However, as is evidenced by the broad diversity of techniques that have been developed to address what are effectively the same set of pre-processing problems, there is no consensus as to how best approach them. It is generally very difficult to provide convincing theoretical arguments for choosing one pre-processing method over others, as essentially none of them are derived from the first principles of the LC/TOFMS operations. Rather, they are heuristic methods, which are constructed based on an intuitive but rather qualitative understanding of the system to which they are applied.

These heuristic methods are often validated by means of direct demonstrations that they produce “reasonable” results when applied to real data, or by arguments that they approximate the steps that would be taken by a trained expert through a more manual analysis [4]. Evaluation of the relative performance of these techniques is extremely difficult as it can be strongly dependent on user experience and the choice of parameters [10, 11]. While there have been calls for the use of “standard data-sets” to compare methods specifically for retention time alignment [11], a meaningful performance comparison based on this approach is likely to remain challenging. Moreover, even if the performance of one method could be established as being consistently better than that of others, its effects on the data and on downstream analyses would remain unclear.

The rationale for employing heuristic techniques in the first place is very rarely articulated, possibly because it is taken for granted that the fundamental operations of mass spectrometers are too complex and involve too many unknown variables to allow for a manageable mathematical description. This concern is not unreasonable in view of the elaborate engineering featured in modern mass spectrometers and the rather limited efforts that have so far been made at developing detailed mathematical models to describe the data they produce. The problem is further compounded by the rapidly evolving design of mass spectrometers and the fact that the vast majority of those used for biological research are commercial models, which renders their precise design details somewhat inaccessible to many researchers in academia. However, a method developed from first principles does not have to account exactly for every aspect of the underlying mass spectrometer design in order to be useful, since approximate models often form a sufficient basis from which to draw the relevant inferences—in the words of George E. P. Box, “all models are wrong but some are useful” [12].

In the following we work from first principles to develop a mathematical model that approximates the underlying probability distribution that governs the raw data produced in an LC/TOFMS experiment. We demonstrate how such a model may in principle be used to address a very wide range of problems central to the analysis of LC/TOFMS data, by means of the traditional tools of classic frequentist statistics. Thus, inferences are made by means of tests of hypotheses, which relate directly to the researcher’s aversion to false positives, and parameter estimates are obtained by means of the method of maximum likelihood. Detailed applications of the approach are outside the scope of this article and we leave them to future publications. Due to uncertainty regarding the nature of mass and chromatographic peaks and of the detection system used, the model breaks down at high ion counts. However, we argue that further refinements in our ability to characterize these fundamental features of the data mathematically could allow us to overcome this restriction and lead to substantial improvements in our ability to analyze and interpret LC/TOFMS data.

## Theory

### Background

As will be discussed below, the data produced by an LC/TOFMS system are in a fundamental sense random and must therefore be described by means of a probability distribution. The model that will be derived in the following approximates this probability distribution (which is extremely complex) by means of a series of binomial distributions. In doing so, it builds on the work of P. B. Coates, who developed methods for the correction of detector saturation, first in the context of radiative lifetime measurements [13] but later also applied to TOFMS data [14]. While the model used by Coates is reminiscent of a binomial distribution, it was never explicitly defined as such, and the assumptions required for its validity were not defined. Moreover, it was used strictly to enhance the effective dynamic range—no attempt was made at relating the model to the mass and chromatographic peak shapes, or to use it to draw broader inferences about the data.

While numerous other models of LC/MS data have been proposed [15–18], there are, to our knowledge, no others that have been developed from the fundamental characteristics of the instrumentation employed. Moreover, we are not aware of any other models whose predictions have been validated in a statistically rigorous manner—model validity is widely argued by means of simple qualitative comparisons to real data.

### The Chromatographic Dimension

We consider a molecular species, *S*, which passes through the chromatographic column, is ionized through electrospray ionization, and accelerated orthogonally onto a detector plate. Our aim is to express the probability of recording a given set of ion counts over the specified mass and retention time ranges.

Owing to the chromatographic separation, the concentration of *S* at the end of the chromatographic column will vary as a function of the retention time, *τ*, in correspondence with the familiar chromatographic peak. It is useful to describe this varying concentration by means of a normalized function, *Γ*(*τ*), which integrates to 1. Thus, if *n* ^(*S*)^ is the total number of molecules of *S* in the mixture, the number of molecules of *S* that elute between the retention times *τ*_*a*_ and *τ*_*b*_ can be expressed as

1 $$ n_{{{\tau_a},{\tau_b}}}^{{(S)}} = {n^{{(S)}}}\int_{{{\tau_a}}}^{{{\tau_b}}} {\Gamma \left( \tau \right)d\tau } $$

and if *p* ^*ionize*^ is the probability that a given molecule of *S* is ionized, the mean number of ionized molecules, *h*, will be given by

2 $$ h = {p^{{ionize}}}{n^{{(S)}}}\int_{{{\tau_a}}}^{{{\tau_b}}} {\Gamma \left( \tau \right)d\tau } . $$

The precise number of molecules that are ionized is governed by the Poisson distribution, for which the probability of obtaining the count, *k*, when the mean count is *h*, is given by

3 $$ P(k) = \frac{{{h^k}{e^{{ - h}}}}}{{k!}}. $$

The Poisson distribution often arises when the probability of an event occurring is very low (the probability that a given molecule is ionized, *p* ^*ionize*^) but has many opportunities to occur (the large number of molecules exiting the chromatographic column). Since *Γ* varies over time, the technical name for the above distribution is a “non-homogeneous Poisson process.” It is noted that although *p* ^*ionize*^ can typically be assumed to be constant over time for a given compound, this will not be the case if its concentration is very high, if a coeluting molecular species causes ionization suppression, or if the ionization is unstable. If such phenomena occur, our model would begin to break down; however, results presented below, along with those from an earlier publication by the authors [19], suggest that such effects are not common, or severe enough, to significantly confound a statistical analysis of the type we aim to perform.

The signal-to-noise ratio of the Poisson distribution increases with the square root of the mean, so that the stochastic nature of the measurement process might in principle become less relevant at high ion counts. However, since current ion counting detection systems get saturated at these high counts and since many important compounds are only present in very low abundances, a purely deterministic model of the data produced is not appropriate. Thus, any model that aims to account for the fundamental uncertainty inherent in LC/TOFMS data must use the Poisson distribution as the basis for doing so.

Current methods of ion transmission are not perfect and many of the ions generated are lost on their way to the orthogonal accelerator (oa), lost following the orthogonal acceleration, or fail to get registered by the detector. However, if the process by which the ions are lost can be regarded as the outcome of a binomial distribution, the distribution of the remaining ion count remains Poissonian.

We will tailor our model to an instrument that injects the ion beam continuously into the oa; however, the same model can be applied with minor modifications to instruments employing an ion trap prior to the oa. If it is assumed that the ion optics do not significantly distort the distribution of the ions of *S*, so that *Γ* may be used to describe the “concentration” of the ions of *S* in the oa a short time after their formation, we can express the mean number of ions there as:

4 $$ h = {p^{{ionize}}}{p^{{oa}}}{n^{{(S)}}}\int_{{{\tau_a}}}^{{{\tau_b}}} {\Gamma \left( \tau \right)d\tau } $$

where *p* ^*oa*^ is the (binomial) probability that a given ion of *S* eventually enters the oa, and *τ*_*a*_ now denotes the time at which the ion beam first enters the oa, and *τ*_*b*_ is the time at which the electric field is applied. It is reasonable to suppose that *p* ^*oa*^ will be constant over time for a given compound unless the ion count is so high that the ions interact significantly with each other; *p* ^*oa*^ may exhibit dependence on other factors, such as the *m/z* value of *S*, but we do not need to know the nature of this dependence for the purposes for which the model will be used.

### The Time-of-Flight Dimension

While we continue to describe the distribution of the ions in time, it is useful to regard the time-of-flight as a separate dimension to the retention time. Thus, while *Γ*(*τ*) describes the relative concentration of *S* as a function of retention time, we now require a function, *Ω*(*t*) to describe the variations in the relative “concentration” of ions at the detector plate as a function of time-of-flight, *t*. In addition to the mass of *S*, the shape of *Ω* reflects factors such as the initial velocity and spatial distributions of the ions at the time the electric field is applied, as well as the strength of the applied field and the length of the flight path. However, for the sake of conciseness, it is written only as a function of the time-of-flight. Factors directly related to the initial ionization, such as matrix effects, are not likely to have much impact on the peak shape due to the collisional cooling that precedes the oa.

The clock that measures the time-of-flight has limited time resolution and thus measures finite time increments of 10s to 100 s of picoseconds for modern TOFMS systems. If a given such interval runs from *t*_*a*_ to *t*_*b*_, the number of ions that arrive at the detector plate over this period remains Poissonian. The absolute number of ions in the oa at *t* = 0, when the electric field is first applied is given by equation 4. If *p* ^*tof*^ is the binomial probability that a given one of these ions strikes an active area of the detector plate, the probability of obtaining *k* ion arrivals in the interval [*t*_*a*_, *t*_*b*_] is given by the Poisson distribution with rate parameter

5 $$ h = {p^{{ionize}}}{p^{{oa}}}{p^{{tof}}}{n^{{(S)}}}\int_{{{\tau_a}}}^{{{\tau_b}}} {\Gamma \left( \tau \right)d\tau } \int_{{{t_a}}}^{{{t_b}}} {\Omega (t)dt} $$

where we disregard detector saturation for now. We have again assumed that *p* ^*tof*^ is constant over time and that *Ω* is independent of the number of ions transmitted, although this requirement would certainly break down at very high ion counts owing to space charge effects [20]. A method for accounting for variations in the shape of *Ω* resulting from the changing intensity of mass peaks, even when the exact nature of this relationship is not known, will be discussed later.

### Ion Detection and Recording

There are a number of separate components that make up the detection system of a TOFMS. The detector itself is an electron multiplier (such as a microchannel plate or a discrete dynode electron multiplier), which amplifies the signal induced by the incoming ion and passes it on to the data acquisition system. TOFMS data acquisition systems employ either an analog-to-digital converter (ADC) or a time-to-digital converter (TDC) in order to convert the electronic signal induced by the electron multiplier into a digital representation of the corresponding time-of-flight [21]. For a single ion injection ADCs have greater dynamic range than TDCs, but they are sensitive to the variable gain of the electron multiplier and to electronic noise, which can be effectively blocked out by TDCs [22]. TDCs also have a higher time resolution so that a better sampling of the mass peaks can be obtained.

TDCs are fundamentally binary, in the sense that in a given increment of the clock they cannot distinguish between the arrival of single and multiple ions. This is part of the reason for their limited dynamic range, but it is also what makes them robust to the variable gain of the electron multiplier. When the rate of ion arrivals is low, TDCs are highly attractive from the point of view of statistical modeling as they can effectively preserve the Poisson distribution of the ion arrivals. A model describing data digitized by means of an ADC would have to account for the electronic noise as well as the uncertainty introduced by the gain of the electron multiplier. For this reason, we will in the following focus on the development of a model tailored to TDC data.

### Histogramming of Binary TDC Data

Once an electronic signal has been digitized by a TDC, it is represented as a binary sequence, indicating only whether zero, or one, or more ions were detected in each of the time increments or “ticks” of the TDC clock. Consequently, these data are not Poissonian, but may be regarded as the outcome of Bernoulli trials where the probability of success is the probability that one or more ions are detected in the tick. Thus, we must make use of a mapping that is also involved in Coates’ correction:

6 $$ P\left( {k \geqslant 1} \right) = 1 - P\left( {k = 0} \right) = 1 - {e^{{ - {p^{{ionize}}}{p^{{oa}}}{p^{{tof}}}{n^{{(S)}}}\int_{{{\tau_a}}}^{{{\tau_b}}} {\Gamma \left( \tau \right)d\tau } \int_{{{t_a}}}^{{{t_b}}} {\Omega (t)dt} }}} $$

where *t*_*a*_ and *t*_*b*_ are chosen such that they define a time-of-flight interval corresponding to a tick of the TDC clock. For the mass spectrometer used in this study, this tick lasts for 250 ps, but more recent instruments use TDCs with acquisition rates as low as 25 ps [23].

The acceleration of a single group of ions from the oa onto the detector plate (a pulse) can take place in a very short period of time, so that a very large number of pulses are acquired over the course of a typical 20-min LC/TOFMS experiment. In order to make the resulting data-set more easily interpretable and reduce it in size, matching time increments from hundreds to thousands of consecutive pulses are summed or “histogrammed” in memory to give rise to the familiar mass spectra. This histogramming, which transforms a sequence of, say, *N*_*p*_ pulses into a single “scan,” is one of the most fundamental forms of pre-processing that is applied to TOFMS data, but in view of current computational limitations it is not one that can be foregone.

If *Γ* is approximately constant across the pulses that are histogrammed, and if the length of the flight path and power supply output are sufficiently stable over the corresponding period of time that *Ω* remains approximately constant over matching ticks in distinct pulses, the Bernoulli trials can be considered to be independent and identically distributed. Consequently, the counts obtained by histogramming the pulses may be regarded as the outcome of a binomial distribution. In view of the short period of time involved and the comparatively modest slope of *Γ*, the assumptions (which are also required for Coates’ correction) are not unreasonable. The “scan time” over which pulses are histogrammed is typically less than 0.01 s for the mass spectrometer used in this study and will be labeled *τ*_*ε*_ in the following.

In order to express the relationship between the underlying Poisson rate parameter and the final binomial probability, we must introduce one further term related to the sampling of the ion beam in the oa. A substantial delay is required following the application of each pulse in order to ensure that the heaviest (and therefore slowest) of the ions has reached the detector plate prior to the application of the next pulse. Consequently, *Γ* is not sampled over contiguous time intervals over the course of a scan, and there is systematic discrimination against low-*m/z* ions as these have higher velocities and, therefore, traverse the oa faster. However, the number of ions lost in this manner may yet again be accounted for through the introduction of a binomial probability, *p* ^*scan*^, which indicates the probability that a given ion is in an axial segment of the ion beam that gets accelerated out of the oa rather than a segment that passes through it. Given the typically high pulsing frequency relative to the slope of *Γ*, *p* ^*scan*^ may be regarded as being independent of the precise shape of *Γ* and, therefore, as being constant over time. It is noted that many modern TOF mass spectrometers incorporate an ion trap prior to the oa where incoming ions can accumulate while the preceding pulse is completed. This greatly improves the duty cycle so that *p* ^*scan*^ can be close to 1 for such instruments, though it may also cause further distortions to the shape of *Γ*.

We can now write the total expected ion count, *I*, over the entire peak in both dimensions as

7 $$ I = {n^{{(S)}}}{p^{{ionize}}}{p^{{oa}}}{p^{{tof}}}{p^{{scan}}} $$

and we can write the Poisson rate function, which describes the variation in mean ion intensity in both the time-of-flight and retention time dimensions as

8 $$ \lambda \left( {\tau, t} \right) = I\Gamma \left( \tau \right)\Omega (t) $$

though it is stressed that *Ω* and *Γ* will in general exhibit a certain dependence on at least some of the factors of *I*.

It is furthermore useful to define a discretized rate function, *Λ*_*i,j*_, that specifies the mean ion count over all the pulses associated with the *i*th chromatographic scan at the *j*th tick, where *i* and *j* are preferably chosen with reference to the first scan and the first tick in which *S* is observed:

9 $$ {\Lambda_{{i,j}}} = \int_{{{\tau_i}}}^{{{\tau_i} + {\tau_{\varepsilon }}}} {\int_{{{t_j}}}^{{{t_{{j + 1}}}}} {\lambda \left( {\tau, t} \right)dt} \;d\tau = } I\int_{{{\tau_i}}}^{{{\tau_i} + {\tau_{\varepsilon }}}} {\Gamma \left( \tau \right)d\tau \int_{{{t_j}}}^{{{t_{{j + 1}}}}} {\Omega (t)dt} } . $$

We can then express the final binomial probability, *ρ*_*i,j*_, of obtaining one or more ion counts in a given pulse of the *i*th chromatographic scan, at the *j*th time-of-flight tick. Since there are *N*_*p*_ such pulses and *Γ* has been assumed to be approximately constant over them, this is written:

10 $$ {\rho_{{i,j}}} = P\left( {{k_{{i,\,j}}} \ge 1} \right) = 1 - P\left( {{k_{{i,\,j}}} = 0} \right) = 1 - {e^{{ - {{{{\Lambda_{{i,\,j}}}}} \left/ {{{N_p}}} \right.}}}}. $$

So that the probability of obtaining a count of *k*_*i,j*_, at the *i*th scan and *j*th time-of-flight tick, is given by

11 $$ P{\left( {k_{{i,j}} } \right)} = {\left( {\begin{array}{*{20}c} {{N_{p} }}  \\  {{k_{{i,j}} }}  \\    \end{array} } \right)}\begin{array}{*{20}c} {{k_{{i,j}} }}  \\   {{\rho _{{i,j}} }}  \\  \end{array} {\left( {1 - \rho _{{i,j}} } \right)}^{{N_{p}  - k_{{i,j}} }} . $$

### Dead Time

In addition to the binary truncation, instruments employing TDCs are affected by a substantial period of “dead time” following the detection of an ion, during which they are incapable of detecting further ions. Different dead time effects are caused by different components of the detection system, and the overall dead time depends on the data acquisition strategy used. A simple and commonly used strategy is to record an ion arrival when the voltage signal it induces crosses a certain threshold, so that the voltage has to recede to a level below this threshold before further ions can be recorded. The dead time is then roughly equal to the width of the voltage signal at this threshold and it typically lasts for a time period on the order of a few ns (around 5 for the mass spectrometer used in this study [24]) so that it spans several of the TDC time increments. It is primarily of the “extending type” so that further ion arrivals during the dead time period will extend it further [25]. A more elaborate method of data acquisition involves the use of constant fraction discriminators, which record the ion arrival when the signal exceeds a specified fraction of the maximum signal height. This provides a more consistent response to the variable output of the electron multiplier, although the dead time will tend to be longer [26]. The dead time is the primary cause of detector saturation for TDC-based mass spectrometers and many methods of reducing its effects and thereby improving the dynamic range have been explored. These include statistical corrections [13, 14, 25], attenuating the beam of incoming ions [6], and using detection systems with multiple anodes and multi-channel TDCs, which allow for the independent recording of distinct ion arrivals [27, 28].

**Figure 1:**
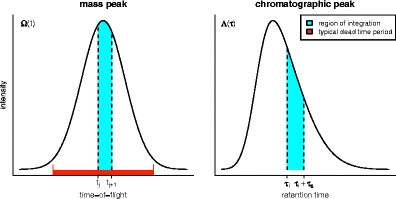
Two “slices’” of the Poisson rate function, *λ*, illustrating its shape in the time-of-flight and retention time dimensions. The region of the rate function that is integrated when calculating *Λ*_*i,j*_ is highlighted in cyan and a representative dead time period is indicated in red

For many mass spectrometers, the time-of-flight “width” of the mass peaks is of the same order as the dead time period [29]. This greatly facilitates the modeling problem as it makes it easier for us to account for how many of the *N*_*p*_ pulses are “valid” in each of the time-of-flight ticks, that is, how many of them are capable of registering further ion arrivals. Only the pulses that are “closed” at the leading edge of the mass peak are likely to recover from the dead time over the duration of the mass peak. If the rate of ion arrivals is moderate, it is unlikely that another ion will strike the detector at this point. If it is high, and the dead time is of the extending type, it is likely that the dead time period would have been extended to cover the remaining mass peak by other incoming ions prior to the expected recovery. This can be illustrated with some simple simulations. We simulate the total number of ion arrivals over a pulse with the Poisson distribution and we simulate the arrival time of each of these ions based on a Gaussian peak shape and investigate whether the largest difference in ion arrival time exceeds the dead time, which we take to be three standard deviations. If the Poisson rate is taken to be 0.5 (moderate), only 2735 out of 10^6^ pulses (0.27%) had arrival time differences greater than the dead time; for a Poisson rate of 5 (high), 8037 out of 10^6^ pulses (0.80%) had arrival time differences greater than the dead time. Thus, we will assume that when a pulse is closed by an incoming ion, it remains so for the duration of the mass peak. This model is somewhat simplistic as it ignores the detailed reality of the behavior of the electronics, but it provides an adequate first approximation. A schematic illustration of mass and chromatographic peak shapes and of the dead time period is given in Figure 1.

If *k*_*i,j*_ denotes the ion count observed in the *i*th scan and at the *j*th tick of the TDC, then assuming all *N*_*p*_ pulses are valid at time-of-flight *t*_*1*_ where the mass peak “starts”, we can write the number of valid pulses at the *j*th tick, *V*_*i,j*_, as

12 $$ {V_{{i,\,j}}} = {N_p} - \sum\limits_{{x = 1}}^{{x = j - 1}} {{k_{{i,x}}}} $$

so that the recorded ion count at the *j*th tick adheres to the binomial distribution:

13 $$ P{\left( {k_{{i,j}} } \right)} = {\left( {\begin{array}{*{20}c} {{V_{{i,j}} }}  \\ {{k_{{i,j}} }}  \\   \end{array} } \right)}\begin{array}{*{20}c} {{k_{{i,j}} }}  \\    {{\rho _{{i,j}} }}  \\  \end{array} {\left( {1 - \rho _{{i,j}} } \right)}^{{V_{{i,j}}  - k_{{i,j}} }} . $$

In practice, when applying the above model to real data it is best to do so over a short time-of-flight period of similar or of shorter duration than the dead time period, to help ensure the validity of the above assumption. Mass peaks have been observed, which have tails that are heavier than Gaussian [30] and which, therefore, have a higher probability of containing successive ion arrivals that are farther apart than the dead time period. For such peaks, it is not always possible to ensure that the value of *V*_*i,1*_ is exactly equal to *N*_*p*_, but as will be shown, the approximation provided by this model works well for mass peaks of moderate ion counts.

It is straightforward to extend the model to describe multiple chromatographic scans and time-of-flight ticks. Suppose we wish to describe the ion counts of *S* that are recorded over *N* chromatographic scans and *M* time-of-flight ticks. If **k** is a matrix such that *k*_*i,j*_ is the observed ion count in the *i*th scan and the *j*th tick, that is

14 $$ K = {\left( {\begin{array}{*{20}c} {{k_{{1,1}} }} & { \cdots } & {{k_{{1,M}} }}  \\    { \vdots } & { \ddots } & { \vdots }  \\   {{k_{{N,1}} }} & { \cdots } & {{k_{{N,M}} }}  \\ \end{array} } \right)} $$

then taking these counts to be conditionally independent, the probability distribution for the full set of ion counts can be written:

15 $$ P{\left( K \right)} = {\prod\limits_{i = 1}^N {{\prod\limits_{j = 1}^M {{\left\{ {{\left( {\begin{array}{*{20}c}   {{V_{{i,j}} }}  \\   {{k_{{i,j}} }}  \\  \end{array} } \right)}\begin{array}{*{20}c}   {{k_{{i,j}} }}  \\   {{\rho _{{i,j}} }}  \\  \end{array} {\left( {1 - \rho _{{i,j}} } \right)}^{{V_{{i,j}}  - k_{{i,j}} }} } \right\}}} }} } $$

where *V*_*i,j*_ and *ρ*_*i,j*_ are defined as before. This probability distribution will in the following be referred to as the “basic model.”

### Model Limitations

The most demanding assumptions of the basic model are the requirements that the number of valid pulses is equal to *N*_*p*_ at the start of the time-of-flight range and that the length of the dead time invariably exceeds the remaining time-of-flight range. Small deviations from these assumptions do not render the model inapplicable, but it is nevertheless an important respect in which it is incomplete and a key reason why it breaks down at high ion counts. The construction of a more comprehensive model for LC/TOFMS data would require detailed knowledge of the workings of the detector system and, in particular, of the statistical distribution of the dead time. It is also highly likely that other components of the detector system would require more attentive modeling at extreme ion counts.

In addition, the basic model is incomplete in the sense that the functional forms of *Γ* and *Ω* have not been specified and neither has the nature of their dependence on *I*. We stress that the functions in question are those that define the underlying rate function and which, therefore, govern the rate of ion arrivals at the detector plate. We are not referring to the shapes of the peaks observed in the data, which will have been somewhat distorted by the Poisson noise and by detector saturation. Several papers and patent applications have modeled mass peaks based on a Gaussian shape [18, 25, 31–33], but significant deviations from this functional form have been noted at the tails of mass peaks [30, 33]. A more formalized approach to peak modeling has been presented by Opsal et al. through the use of convolutions of probability densities [34]. However, the shapes of mass peaks are specific to the instrument employed and although many manufacturers do develop detailed models of the mass peaks using ion optics simulation software, such as SIMION [35], these are generally not available to researchers in academia. A number of models have also been developed for chromatographic peaks [36] but, again, no single model has been found to be satisfactory under all circumstances [37]. Obtaining an appropriate functional form for *Γ* might be especially difficult since any distortions to the chromatographic peak shape resulting from the ion optics or the ionization would have to be accounted for. Until these fundamental questions in the theory of chromatography and the theory of time-of-flight mass spectrometry have been more comprehensively answered and disseminated to researchers in academia, important constraints on the statistical modeling of LC/TOFMS data are inevitable. Nonetheless, knowledge of *Γ* and *Ω* is not required for the basic model to be of use. By working with the discretized rate function, *Λ*_*i,j*_, it is possible to obtain estimates of the true rate of ion arrivals, irrespective of the functional forms of *Γ* and *Ω*. However, this requires a total of *NM* estimates to fully characterize the rate function. For the mass spectrometer used in this study, *M* is typically between 10 and 20, while *N* can range from around 10 to several 100 for chromatographic peaks with heavy tailing. Whatever the true functional forms of *Γ* and *Ω* are, they will undoubtedly require far fewer parameters, so that there are effectively more data available for each parameter that must be estimated to fully describe the rate function if one or both functions are known. Furthermore, the construction of statistical tests based on the basic model is greatly facilitated if *Γ* and *Ω* are known.

### Estimation and Inference Using the Basic Model

Although *Γ* and *Ω* are described as being functions of the time-of-flight and the retention time, they depend on a larger number of parameters. Some of these are likely to be nuisance parameters that provide little information on *S*, but clearly *μ*_*Ω*_—the location parameter of *Ω*—is of great interest as it relates to the mass of *S*. Other parameters must include, at a minimum, a scale parameter for *Ω*: *σ*_*Ω*_ , and location, scale, and skewness parameters for *Γ: μ*_*Γ*_, *σ*_*Γ*_ and *γ*_*Γ*_ respectively. The parameters of *Γ* may exhibit dependence on *n* ^*(S)*^ and those of *Ω* may be affected by the intensity of the mass peaks, but this dependence may be limited for moderate analyte abundances and ion counts.

When the probability distribution of the acquired data is known, the problem of parameter estimation may be addressed by means of the widely used method of maximum likelihood. Note that the left-hand side of equation 15 should, strictly speaking, be written P(**k** | *μ*_*Ω*_ , *σ*_*Ω*_ *, μ*_*Γ*_, *σ*_*Γ*_, *γ*_*Γ*_, *I*, *N*_*p*_) in order to make explicit the dependence of the probability distribution on the parameters of the system. However, we can reinterpret this probability distribution as the likelihood function L(*μ*_*Ω*_ , *σ*_*Ω*_ *, μ*_*Γ*_, *σ*_*Γ*_, *γ*_*Γ*_, *I* | **k** , *N*_*p*_), for which we allow the parameters to vary but consider **k** (and *N*_*p*_, which is always known) to be fixed. We then find the parameters that maximize the likelihood function and use these as our estimates as they are the ones that would give rise to the observed data with the highest possible probability. Clearly, this approach has a stronger theoretical appeal than a simple centroid, or even a least-squares fit; moreover it implicitly corrects for the effects of histogramming and dead time as these are incorporated into the model. If the shapes of *Γ* and *Ω* are not known, we may instead write out the likelihood as a direct function of all *NM* individual *Λ*_*i,j*_ and find maximum likelihood estimates of these.

But perhaps more importantly, the basic model may be used to draw a broad range of inferences by means of general statistical tests such as the likelihood ratio test. The basic model can be used to describe a wide range of features of the data acquired in LC/TOFMS experiments by expanding the likelihood function accordingly. Certain hypotheses that the analyst may have regarding the acquired data can be expressed very naturally by placing specific constraints on the likelihood function, and thereby reducing the total number of parameters of the model. The likelihood ratio test can be used to assess whether such hypotheses are plausible, by determining whether or not the unconstrained model is significantly better at describing the acquired data than the constrained one is.

More specifically, suppose *L*_*0*_ is the supremum of the likelihood function for the constrained model, and *L*_*A*_ is the supremum for the unconstrained one, and let *d* be the difference in dimensionality of the two models. If the hypothesis is true, and the constraint is appropriate, then under certain regularity conditions for the likelihood functions, it can be shown [38] that for large sample sizes:

16 $$ {X^2} = - 2\log \left( {\frac{{{L_0}}}{{{L_A}}}} \right)\sim \chi_d^2. $$

That is, the *X* ^2^ statistic adheres to a *χ* ^*2*^-distribution with *d* degrees of freedom. Thus, by comparing *X* ^2^ to the cumulative distribution function of the appropriate *χ* ^*2*^-distribution, we can determine whether the data are consistent with the hypothesis associated with the constrained model at a given significance level. Given that this result generally requires large sample sizes, care must be taken to ensure that it applies in practice.

An important practical difficulty in applying the likelihood ratio test lies in finding *L*_*0*_ and *L*_*A*_ in the first place. Since the likelihood functions encountered in this study are quite complex, analytical solutions are not generally available and, consequently, numerical methods must be employed. Aside from the inevitable computational demands this entails, caution must be exercised to ensure that the errors associated with the final approximations are very small relative to values typical of the *χ* ^*2*^_*d*_-distribution.

### Validation Procedure

The validity of the basic model may be tested by determining whether its predictions are borne out in real LC/TOFMS data. For this purpose we will consider the phenomenon of fragmentation, where some molecules of *S* (the parent molecule) break up into a smaller molecular species, *R* (the fragment), which may also get detected by the mass spectrometer. In practice, the task of identifying related parent-fragment pairs is extremely important as it can provide vital structural information on *S*. However, it is often confounded by the fact that compounds unrelated to *S* can elute from the chromatographic column at roughly the same time.

Since the fragmentation takes place following the elution of *S* from the column, the chromatographic peak shapes of *S* and *R* should be identical, that is *Γ* ^*(S)*^ = *Γ* ^*(R)*^. In contrast, the chromatographic peak shapes of unrelated compounds are likely to be quite distinct, even when they elute at roughly the same time. We refer to these two scenarios as “exact coelution” and “partial coelution,” respectively. In the framework of the likelihood ratio test, which we will apply to parent-fragment pairs observed in real data, the unconstrained model corresponds to partial coelution, and the constrained one corresponds to exact coelution. The authors have previously proposed a test of hypothesis for exact coelution [19], however, that was for a much more idealized model intended strictly for centroided data, in which the distribution of the recorded ion counts was assumed to be close to Poissonian. Thus, in addition to validating the basic model, we will in the following provide a more general, albeit more computationally demanding, test of hypothesis for identifying parent-fragment pairs.

The Gaussian function is regarded as a reasonable, but imperfect model for the shape of the underlying mass peak. Despite its shortcomings, we will use this model in our validation procedure so that

17 $$ {\int_{t_{j} }^{t_{{j + 1}} } {\Omega {\left( t \right)}dt = \frac{1} {{{\sqrt {2\pi \sigma ^{2}_{\Omega } } }}}{\int_{t_{j} }^{t_{{j + 1}} } {e^{{ - \frac{{{\left( {t - \mu \Omega } \right)}^{2} }} {{2\sigma ^{2}_{\Omega } }}}} } }dt.} } $$

If the Gaussian shape is in fact a poor model for describing the underlying mass peaks, the likely effect will be a rejection of the validity of the basic model, and so this assumption only serves to make the validation procedure more conservative. If the validation proves successful, we will have direct evidence that the Gaussian mass peak model, despite its faults, is sufficiently accurate to allow for the construction of statistical tests, which is the only central requirement of the model for the purposes of this study.

We will leave *Γ* unspecified, so that

18 $$ I\int_{{{\tau_i}}}^{{{\tau_i} + {\tau_{\varepsilon }}}} {\Gamma \left( \tau \right)d\tau } = {I_i} $$

where the “intensity factors,” *I*_*i*_, must be estimated independently for each chromatographic scan. For the purpose of validating the basic model, we will also fit *μ*_*Ω*_ and *σ*_*Ω*_ independently at each scan. This is primarily to simplify the maximization of the likelihood function, which in turn must be done independently for each chromatographic scan. However, it also accounts for potentially confounding effects, for example that the values of *μ*_*Ω*_ and *σ*_*Ω*_ might exhibit dependence on the intensity of the mass peak so that *Ω* and *I* are not independent, or that *μ*_*Ω*_ might drift over time due to temperature fluctuations. The discretized rate function of *S* is then written

19 $$ \Lambda ^{{{\left( S \right)}}}_{{i,j}} = I^{{{\left( S \right)}}}_{i} \frac{1} {{{\sqrt {2\pi \sigma ^{2}_{{\Omega ,i}} } }}}{\int_{t_{i} }^{t_{{i + 1}} } {e^{{ - \frac{{{\left( {t - \mu _{{\Omega ,i}} } \right)}^{2} }} {{2\sigma ^{2}_{{\Omega ,i}} }}}} } }dt. $$

In fitting the likelihood function to a mass peak at a given scan, two parameters are required for the Gaussian shape and one for the intensity factor. Consequently, fitting the model to all of the mass peaks obtained over a chromatographic peak of *N* scans requires 3 *N* parameters.

In the scenario of partial coelution, where *S* and *R* have distinct chromatographic peak shapes, the likelihood function is fitted to the two sets of mass peaks independently and, therefore, 3 + 3 = 6 parameters are required for a single chromatographic scan and 6 *N* for the full data-set. For exact coelution, the basic model must be fitted to *S* and *R* with the constraint that their chromatographic peak shapes are identical. Consequently, the ratio of their intensity factors can be written

20 $$ \frac{{I_i^{{(S)}}}}{{I_i^{{(R)}}}} = \frac{{{I^{{(S)}}}\int_{{{\tau_i}}}^{{{\tau_i} + {\tau_{\varepsilon }}}} {\Gamma \left( \tau \right)d\tau } }}{{{I^{{(R)}}}\int_{{{\tau_i}}}^{{{\tau_i} + {\tau_{\varepsilon }}}} {\Gamma \left( \tau \right)d\tau } }} = \frac{{{I^{{(S)}}}}}{{{I^{{(R)}}}}} = b $$

which is constant across all scans of the chromatographic peaks if, as has been argued in the derivation of the basic model, the various binomial factors of *I* ^*(S)*^ and *I* ^*(R)*^ are constant over time. Therefore, the basic model must be fitted to *S* and *R* simultaneously, using the constraint that *I*_*i*_^*(S)*^ = *bI*_*i*_^*(R)*^ for each chromatographic scan. Consequently, we require effectively only five parameters to fit the likelihood function to a single scan, and 5 *N* + 1 for the full data-set, the extra parameter being *b*. Provided *N* is sufficiently large, a satisfactory estimate of *b* can be obtained by taking the median of the ratios provided by the estimates of the intensity factors obtained from the unconstrained model.

For a given mass peak, the difference in the number of parameters for the constrained and the unconstrained models is effectively 1, and for *N* scans the difference is *N* – 1. Thus if the basic model provides a good approximation to the true distribution of the empirical data, applying the likelihood ratio test to known parent-fragment pairs should give rise to *X* ^*2*^ statistics that are distributed according to the *χ* ^*2*^_*1*_-distribution for individual scans and the *χ* ^*2*^_*N–1*_-distribution for the full data-set. By comparing the empirical values of the *X* ^*2*^ statistics to the cumulative distribution function of the appropriate *χ* ^*2*^-distribution, a *P* value can be obtained, indicating whether or not the data are consistent with this null hypothesis. A similar type of validation procedure, based on a comparison of predicted and theoretical test statistics, was used by the authors for the simple Poisson model of centroided counts [19].

## Experimental

The only critical requirements of the data used for the validation procedure described above is that they are raw, that genuine fragment pairs are used, and that these are not “contaminated” by coeluting compounds with similar *m/z* values. To help ensure this, a mixture of known metabolites that has previously been studied by the authors was used for the analysis.

### Sample Preparation

Eighty-three mammalian metabolites were weighed into a 1 L bottle and dissolved in 1 L HPLC grade water (Sigma-Aldrich, St. Louis, MO, USA). All remaining solids were removed through vacuum filtration. The metabolite concentrations were targeted to fall between 1 and 20 mM, and sodium azide was added at 0.05% vol/vol as a preservative. The stock solution was stored at –80 ºC. In addition to the original sample, 2-, 10-, and 20–fold dilutions were prepared.

### Instrumentation

The samples (5 μL) were injected onto a 2.1 × 100 mm (1.7 μm) HSS T3 Acquity column (Waters Corporation, Milford, MA, USA) and eluted with a 18 min gradient of 100% A to 100% B (A = water, 0.1% formic acid, B = acetonitrile, 0.1% formic acid). The flow rate was set to 500 μL/min, the column temperature 40 ºC and the sample temperature 4 ºC. The samples were analyzed using a UPLC system (UPLC Acquity, Waters Ltd. Elstree, UK) coupled online to a Q-TOF Premier mass spectrometer (Waters MS Technologies, Ltd., Manchester, UK) in positive and negative ion electrospray mode with a scan time of 0.08 s and a scan range of 50–1000 *m/z*. Three technical replicates were run for each of the four dilutions. The mass spectrometer was run in continuum mode and the detector saturation correction was switched off. The “DRE lens,” which the Q-TOF Premier uses to minimize detector saturation, was also switched off. We note that the Q-TOF Premier uses a microchannel plate for ion detection, but we anticipate broadly similar results for instruments employing other detector types, provided the associated dead time is comparable to, or longer than, the mass peak widths.

### Data Selection

The likelihood ratio test was applied to the mass peaks of nitrotyrosine, glutaric acid, and hippurate, along with a fragment derived from each of those compounds. The fragments of nitrotyrosine and glutaric acid correspond to a loss of CO_2_ and the fragment of hippurate corresponds to a loss of glycine. Data from all four dilutions and each of the three technical replicates were used, giving a total of 12 data-sets and 24 peaks. The raw counts for each of these peaks were inspected in order to reduce the risk of possible interference from other compounds. While their presence cannot be definitively excluded by this approach, such contamination would tend to distort the distribution of the statistics obtained from the likelihood ratio test, which would lead us to reject the validity of the basic model. In order to assess the effects of detector saturation, the likelihood ratio test was applied to the data over three sets of ion count ranges constituting the tertiles of the full ion count range.

## Results and Discussion

For clarity, we reiterate the steps involved in the calculation of the test statistics. If for the *i*th scan we observe the ion counts *k*_*i,1*_, *k*_*i,2*_, …, *k*_*i,M*_ across one of the mass peaks, we define the corresponding likelihood function as

21 $$ L{\left( {\mu _{{\Omega ,i}} ,\sigma ^{2}_{{\Omega ,i}} ,I_{i} \left| {k_{{i,1}} ,k_{{i,2}} ,...,k_{{i,M}} } \right.} \right)} = {\prod\limits_{j = 1}^M {{\left\{ {{\left( {\begin{array}{*{20}c}   {{V_{{i,j}} }}  \\   {{k_{{i,j}} }}  \\  \end{array} } \right)}\begin{array}{*{20}c}    {{k_{{i,j}} }}  \\    {{\rho _{{i,j}} }}  \\  \end{array} {\left( {1 - \rho _{{i,j}} } \right)}^{{V_{{i,j}}  - k_{{i,j}} }} } \right\}}} } $$

where

22 $$ \begin{array}{*{20}c} {\rho _{{i,j}} = 1 - e^{{ - \Lambda _{{i,j}} /N_{p} }} {\text{and}}\Lambda _{{i,j}} } \\ { = I_{i} \frac{1} {{{\sqrt {2\pi \sigma _{{\Omega ,i}} ^{2} } }}}{\int_{t_{i} }^{t_{{i + 1}} } {e^{{ - \frac{{{\left( {t - \mu _{{\Omega ,i}} } \right)}^{2} }} {{2\sigma ^{2}_{{\Omega ,i}} }}}} } }dt} \\ \end{array} $$

and we find the set of parameters that maximize this likelihood function, and record its value. In the case of partial coelution we apply this procedure independently to the two mass peaks studied and let the full likelihood, *L*_*A*_, be the product of the two individual maximum likelihood values. For exact coelution the two likelihood functions must be maximized simultaneously, and with the constraint that the ratio between the two *I*_*i*_s must equal *b*, as discussed earlier. If *L*_*0*_ is the maximum likelihood for exact coelution, then the test statistic is given by

23 $$ {X^2} = - 2\log \left( {\frac{{{L_0}}}{{{L_A}}}} \right) $$

which should be governed by the *χ* ^*2*^_*1*_-distribution. The whole procedure is repeated for all *N* scans studied, giving a total of *N* test statistics, whose sum gives us a pooled test statistic which should be governed by the *χ* ^*2*^_*N-1*_-distribution.

In addition to testing the validity of the basic model, it is worth investigating whether the quite considerable level of detail that it includes is even necessary. Therefore, the same likelihood ratio test was also constructed for the more parsimonious model, which assumes the ion counts to be purely Poissonian. For this pure Poisson model, the procedure is the same, except that the above likelihood function is given by

24 $$ L\left( {{\mu_{{\Omega, i}}},\sigma_{{\Omega, i}}^2,{I_i}\left| {{k_{{i,1}}},{k_{{i,2}}}, \ldots, {k_{{i,M}}}} \right.} \right) = \prod\limits_{{j = 1}}^M {\left\{ {\frac{{{e^{{ - {\Lambda_{{i,\,j}}}}}}\Lambda_{{i,\,j}}^{{{k_{{i,\,j}}}}}}}{{{k_{{i,\,j}}}!}}} \right\}} . $$

**Figure 2:**
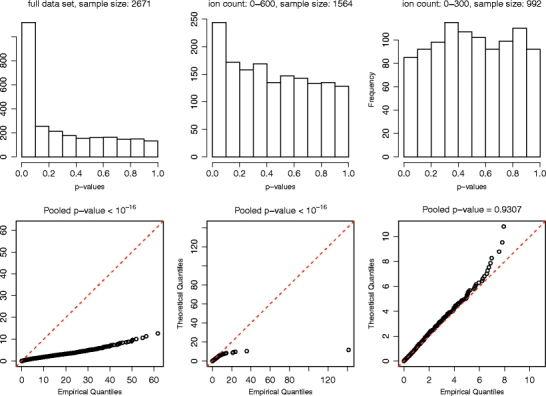
Top: histograms of the *P* values associated with the X^2^ statistics obtained from the individual scans over each of the three ion count ranges by using the pure Poisson model to construct the likelihood ratio test. Bottom: quantile-quantile plots of the X^2^ statistics themselves compared with the theoretical X^2^_*1*_-distribution. The *P* values obtained for the pooled data-sets are listed above the quantile-quantile plots. Only for the lowest range of ion counts do the statistics appear to conform reasonably well to the X^2^_*1*_-distribution predicted by the likelihood ratio test

The likelihood functions were maximized by means of a Newton-type algorithm [39, 40] implemented in the R statistical programming language [41]. This optimization method requires knowledge of the likelihood function, its gradient (the vector of first-order partial derivatives) and its Hessian (the matrix of second-order partial derivatives). The gradient of the likelihood function was calculated analytically and was in turn used to obtain numerical estimates of the Hessian. After convergence, several of the likelihood functions were visually inspected in all dimensions near the maximum likelihood estimates in order to help ensure that a maximum had indeed been attained. The results for the pure Poisson model are shown in Figure 2, where quantile–quantile plots [42] are drawn of the *X* ^*2*^ statistics along with histograms of the associated *P* values for each of the three ion count ranges investigated. If the *X* ^*2*^ statistics adhere to the predicted distribution, there should be only moderate deviations from the 45° angle on the quantile–quantile plots (indicated by the red lines), and the *P* values should be uniformly distributed.

The results make good sense. At low ion counts where detector saturation is minimal, the pure Poisson model does a satisfactory job of explaining the observed data and, consequently, the *X* ^*2*^ statistics conform quite closely to the distribution predicted by the likelihood ratio test. But at higher counts, the saturation effects become more substantial and significant deviations from the predicted distribution are evident. There is a slight indication that the *X* ^*2*^ statistics obtained for the lower tertile tend to be marginally smaller than those of the *χ* ^*2*^_*1*_-distribution, and this is consistent with results obtained from simulated data (not shown).

**Figure 3:**
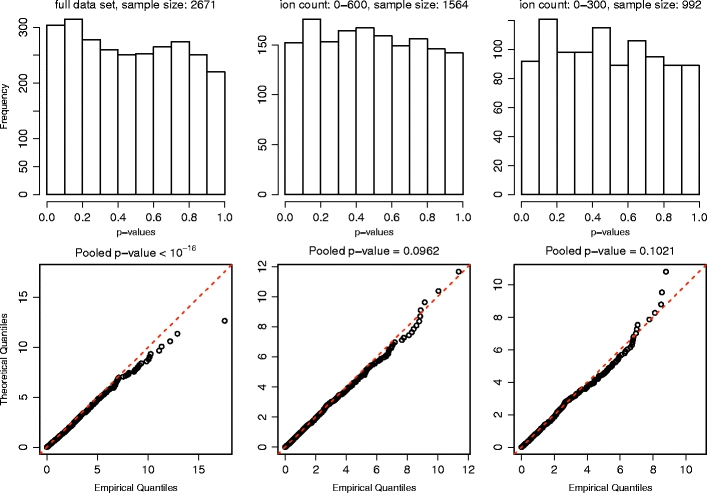
Top: histograms of the *P* values associated with the χ^2^ statistics obtained from the individual scans over each of the three ion count ranges by using the basic model to construct the likelihood ratio test. Bottom: quantile-quantile plots of the χ^2^ statistics themselves compared with the theoretical χ^2^_*1*_-distribution. The *P* values obtained for the pooled data-sets are listed above the quantile-quantile plots. The fit is substantially better than that obtained for the pure Poisson model although the basic model does break down at high ion counts

The results for the basic model are shown in Figure 3. It is clear that the fit obtained is significantly better than that of the pure Poisson model as the *X* ^*2*^ statistics for the individual scans are consistent with the predicted distributions over both of the two lower tertiles of the ion count range. Moreover, when the full ion count range is used, they exhibit deviations that are comparatively very modest. The pooled *P* values from the two lower tertiles are somewhat low; however, we must recall that the large sample size will tend to make the pooled *P* values sensitive even to slight departures from model assumptions and that the Gaussian peak shape does not exactly reflect the true rate function.

Thus, we have strong evidence that the basic model closely approximates the probability distribution of the acquired data when the ion counts are moderate or low and that the mathematical modeling of the saturation effects is not superfluous. In addition, we have a direct demonstration that the likelihood ratio test can be used in practice to make inferences about the sample being analyzed. In view of the quite considerable detail with which the basic model has been formulated of the conservative nature of the validation procedure and the very specific predictions made, these results are very encouraging.

## Conclusion

This article has attempted to establish some of the key concepts required to conduct a rigorous statistical analysis of LC/TOFMS data for mass spectrometers employing TDCs. Although demanding simplifying assumptions were made in formulating the basic model, the *X* ^*2*^ statistics obtained from its application to related fragment pairs through the likelihood ratio test conform closely to the predicted distribution so long as the ion counts are not too high. The basic model’s rather high level of detail does not appear to be unwarranted since the fit of the corresponding statistics obtained for the more parsimonious pure Poisson model of continuum data deteriorates much faster as the ion count is increased. Thus, the basic model illustrates the feasibility of detailed statistical modeling of mass spectrometry data and raises the prospects for the development of a formal statistical methodology to address the problems encountered in their analysis.

While the basic model can, in fact, already be applied usefully to LC/TOFMS data, its utility would be greatly expanded if the functional forms of the mass and chromatographic peaks were known and if a more elaborate model were available to account for how many pulses are valid at a given tick of the TDC. This would allow us to provide statistically rigorous solutions to tasks such as dead time correction, deconvolution of overlapping mass peaks, testing the fit of theoretical isotopic abundance patterns, and perhaps, in the long term, testing the fit of theoretical *m/z* values. Development of these applications is outside the scope of this article and we thus leave them to future publications. In addition, such an enhanced model might enable significantly improved estimates of mass and isotopic abundance patterns through the pooling of measurements across distinct chromatographic scans. We, therefore, believe there are strong grounds for increased inquiry into the fundamentals of these instruments.

It is noteworthy that more extensive efforts have not already been made at extending statistical rigor to the analysis of LC/TOFMS data, given its obvious importance. However, it is plainly the case that most of the statistical methods developed for LC/TOFMS take the heuristic approach, which makes no attempt at accounting for the effects of the data generation and pre-processing. This lack of rigor may stem in part from the highly interdisciplinary requirements of the more rigorous approach, bringing together engineering, statistics, and chemistry, as well as the fact that much of the engineering is currently consigned to industry and, therefore, not easily accessible to scientists in academia.

While the potential benefits of adopting a rigorous approach could be substantial, it is of course not yet known whether the required modeling can be properly completed or whether the computational demands can be sufficiently reduced so that the resulting methods can be applied in a routine manner. But it is quite conceivable that such difficulties could be resolved through engineering efforts. While the convention is for statisticians to develop methods of data analysis that can accommodate the data output by an instrument that has been designed independently by engineers, taking a more integrated approach is not at all unreasonable, and could prove to be highly beneficial. Thus, mass spectrometers might be deliberately designed so that the data produced can more easily be described by a probability distribution and so that the maximum likelihood estimators or, rather, the relevant test statistics, can more easily be obtained. This applies to mass spectrometers other than time-of-flight and indeed to any analytical instruments that produce inexact measurements.

This integrated approach may require a slight shift in our conception of what constitutes a good mass spectrometer. Currently, heavy emphasis is placed on developing mass spectrometers with improved mass accuracy, the improvement often being quantified through descriptive statistics such as the root mean square deviation or the average absolute deviation [43]. Similarly, considerable efforts are made at increasing the resolution and dynamic range of the instruments. But in many situations we are not interested in improving these measures for their own sake—we generally care about them only to the extent that they help us draw inferences about the sample being analyzed. For example, there are very few proteomics or metabolomics studies for which mass determination is the ultimate goal; what we more typically would want to know is “which compounds have a theoretical *m/z* that has a reasonable chance of giving rise to the observed data.” It is, therefore, important to bear in mind that the fundamental measure by which we must judge the quality of mass spectrometers is the range of inferences that we can draw from the data they produce and the ease with which we can do so. Having the means to form a sound statistical argument is a critical and rather minimal aspect of drawing inferences from acquired data. In this sense it is quite possible that a mass spectrometer, which is sufficiently well characterized mathematically so that common data-analytical problems can be addressed with statistically rigorous methods, might be preferred to one that lacks this property even though the latter might seem better by the conventional measures of mass accuracy, dynamic range, and resolution. In brief, through further efforts at modeling the detailed workings of our instruments, we may begin to move beyond relatively superficial descriptive statistics when judging their quality and place greater emphasis on the actual means of inference that they provide. Clearly, we are at a very early stage of this undertaking.
